# A Case of a Coexisting Carcinosarcoma Ex Pleomorphic Adenoma With Langerhans Cell Histiocytosis in the Parotid Gland

**DOI:** 10.7759/cureus.42351

**Published:** 2023-07-24

**Authors:** Nasser M AlMadan, Shatha M Sulaiman, Furat Almayouf, Mohammed Alwhabi, Turki Alquwayz

**Affiliations:** 1 Dentistry, Prince Sultan Military Medical City, Riyadh, SAU; 2 Pathology, Security Force Hospital, Riyadh, SAU; 3 Anatomic Pathology, King Faisal Specialist Hospital and Research Centre, Riyadh, SAU; 4 Anatomic Pathology, Prince Sultan Military Medical City, Riyadh, SAU; 5 Dentistry, King Salman Armed Forces Hospital, Tabuk, SAU

**Keywords:** parotid, lch, langerhans cell histiocytosis, carcinosarcoma ex pleomorphic adenoma, carcinosarcoma

## Abstract

Carcinosarcoma ex pleomorphic adenoma is a rare malignant neoplasm, with most cases reported in the parotid gland. We herein report a case of a 75-year-old male referred to our hospital with a long-standing right parotid lesion that was treated in an outside hospital by a superficial parotidectomy. The patient reported a painful, rapidly enlarging mass following the excision. Histopathological examination showed the proliferation of malignant epithelial and mesenchymal elements with a solid sheet of Langerhans cells admixed with eosinophils. The Langerhans cells were reactive to CD1a and BRAF; hence, a diagnosis of carcinosarcoma ex pleomorphic adenoma with Langerhans cell histiocytosis was given. Complete clinical and radiographic workup showed no other organ involvement. The patient underwent total parotidectomy with adjuvant chemoradiation; however, the tumor progressed and showed lung metastasis. We herein report the first case of a concurrent Langerhans cell histiocytosis with associated carcinosarcoma ex pleomorphic adenoma.

## Introduction

Carcinosarcoma (also called true malignant mixed tumor) comprises both malignant epithelial and malignant mesenchymal components. It is an exceedingly rare tumor of the salivary gland. Most cases arise de novo; however, some patients have a history of a long-standing or recurrent pleomorphic adenoma, a condition described as carcinosarcoma ex pleomorphic adenoma [1]. It is usually diagnosed with a male predilection in the sixth to the eighth decade [2]. The parotid gland is the most commonly reported site, with some cases in the submandibular gland. Patients usually present with a rapidly enlarging mass of a long-standing lesion that presents for a long time, with some cases associated with pain, facial nerve paralysis, and rarely ear pain [2-11]. Radiographically, it shows well-demarcated or irregularly shaped heterogeneous mass with a peripheral enhancement of variable size that can reach up to 9 cm. Additionally, calcification, necrosis, and metastasis to regional lymph nodes could be seen [2-11].

Microscopically, the lesion shows a biphasic tumor component with the epithelial component showing multiple features, including squamous cell carcinoma (SCC), adenocarcinoma not otherwise specified (NOS), or salivary duct carcinoma (SDC). Less reported cases showed spindle cell carcinoma, mucoepidermoid carcinoma, intraductal carcinoma (IDC), and large cell neuroendocrine carcinoma (LCNEC). The mesenchymal component usually shows chondrosarcoma, osteosarcoma, fibrosarcoma, and rhabdomyosarcoma components. The less commonly reported mesenchymal components include undifferentiated pleomorphic sarcoma, liposarcoma, and myofibrosarcoma. Most cases showed a remnant of pleomorphic adenoma with a transition of the tumor from a benign component to an area that shows atypical pleomorphic adenoma to a frank malignant neoplasm. However, some cases show only fibromyxoid stroma or, more commonly, hyaline nodule as the benign component. Extensive necrosis is common with pleomorphism and increased mitosis [2-11]. The benign component usually shows low Ki67 with no reactivity to p53 and Her2. These stains substantially increased in the transition zone and the frank malignant component. On the contrary, E cadherin is retained in the benign area but lost in the malignant component [2,4,7,8,12,13]. One case was reactive with HMGA2 while one peculiar case showed reactivity to HMGA2 and PLAG1 [8,14]. Management includes surgical resection with neck dissection in cases of lymph node involvement followed by adjuvant radiation. Prognosis is guarded, with many cases showing tumor progression with metastasis to distant sites.

Langerhans cell histiocytosis (LCH) is considered the most common histiocytic disease, characterized by the proliferation of langerin-positive histiocytes (CD207+) with infiltration of inflammatory cells. It can be a localized lesion or involve multiple organs, with the latter showing a more aggressive clinical course [15]. Langerhans cells could be seen in association with other neoplasms as a concurrent neoplasm or just as passenger inflammatory cells. To date, two reported cases of LCH in association with the salivary gland [16,17]. We herein report a case of carcinosarcoma ex pleomorphic adenoma associated with Langerhans cell histiocytosis in a 75-year-old male.

## Case presentation

**Figure 1:**
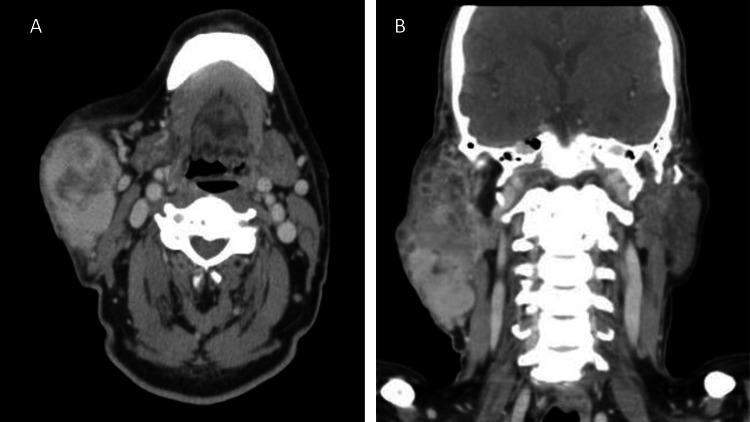
CT scan in axial and coronal views Axial view shows a heterogeneously enhancing and solid cystic lesion (A). Coronal view shows multiple, solid enhancing nodules adjacent to the main lesion (B).

A 75-year-old male was referred to our hospital for a recurrent right parotid mass. The patient reported that the lesion persisted for over 25 years and was slowly enlarging. After that, he received a superficial parotidectomy in an outside hospital. The diagnosis from the referring hospital was pleomorphic adenoma with extensive myxoid degeneration, and they mentioned that the tumor had an involved margin. The patient reported a rapidly enlarging painful mass and was referred to our hospital. The radiological findings showed a large heterogeneously enhancing lobulated solid cystic mass arising from the superficial lobe of the right parotid gland measuring around 10 cm with multiple solid enhancing nodules abutting the primary lesion (Figure 1).

**Figure 2:**
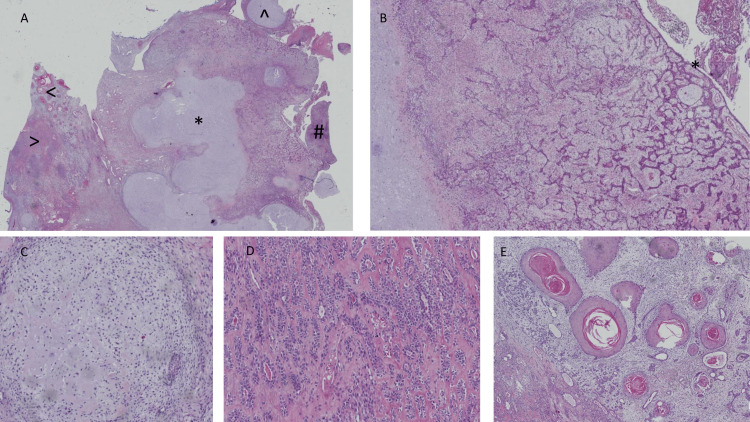
Superficial parotidectomy from the referring hospital for carcinosarcoma ex pleomorphic adenoma with Langerhans cell histiocytosis (A) Low power shows multinodular proliferation with heterogeneous growth with chondromyxoid stroma present in the middle that corresponds to the remaining pleomorphic adenoma (*), malignant chondroid (^), squamous cell carcinoma component (<), malignant glandular proliferation (>) and Langerhans cell histiocytosis (#). (B) Intermediate power shows malignant ductal proliferation surrounded by myoepithelial cells arising from a dysplastic large duct (*). (C) Malignant chondroid. (D) Malignant ductal cells admixed with myoepithelial cells in a hyalinized stroma. (E) Squamous cell carcinoma component.

Histological slides were submitted to our anatomical pathology department. They showed multinodular tumoral tissue with central chondromyxoid stroma corresponding to the remnant of the pleomorphic adenoma component with the proliferation of malignant mesenchymal and epithelial components. The mesenchymal component was composed of malignant chondroid tissue. In contrast, the epithelial component was composed of malignant glandular proliferation surrounded by myoepithelial cells reminiscent of epithelial, myoepithelial carcinoma with an area of squamous differentiation that resembles squamous cell carcinoma (Figure 2).

**Figure 3:**
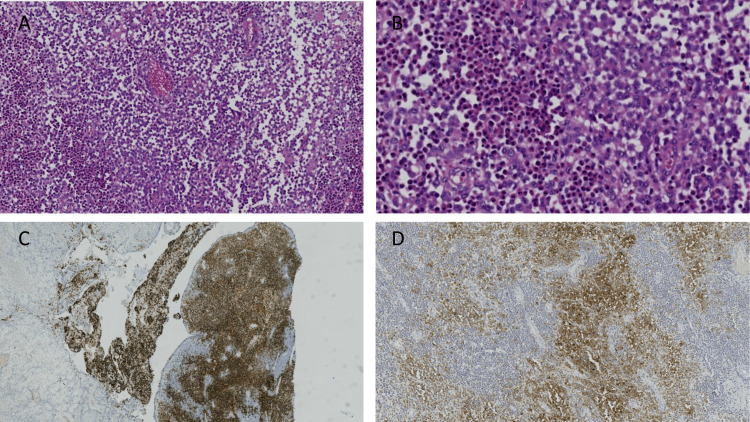
Higher power for the LCH component A proliferation of sheet cells of histiocytes with admixed eosinophils (A), higher power shows histiocytes with coffee-bean nuclei and nuclear grooving (B), diffuse positivity for CD1a (C), nuclear positivity for BRAF IHC (D) LCH: Langerhans cell histiocytosis; IHC: immunohistochemistry

A solid sheet of histiocytes with nuclear grooving admixed with eosinophils was associated with the tumor (Figure 3).

**Figure 4:**
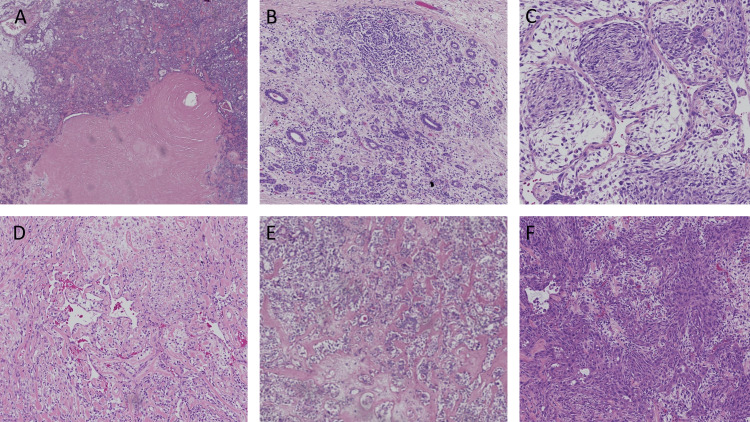
Microscopic feature of total parotidectomy specimen A giant collagen rosette represents the remnant of the pleomorphic adenoma surrounded by malignant glandular proliferation with a remnant of a chondromyxoid stroma in the periphery (A). Lobular proliferation of reactive ducts with acinar atrophy and chronic inflammatory cell infiltrate (B). Whorling of tumor cells (c). Vascular formation between tumor cells (D). Osteoid formation (E). Anastomosing cords of pleomorphic epithelioid cells with tumor giant cells and acantholysis (F).

A carcinosarcoma ex pleomorphic adenoma diagnosis with Langerhans cell histiocytosis was given, and a complete workup was done to rule out systemic involvement. Total parotidectomy with adjuvant chemoradiation was initiated with the excisional specimen showing a similar diagnosis (Figure 4).

Unfortunately, the patient showed tumor progression with lung metastasis following therapy.

## Discussion

Carcinosarcoma is an exceedingly rare tumor of the salivary gland, with the majority of cases arising de novo and some arising from a previous pleomorphic adenoma, a term called carcinosarcoma ex pleomorphic adenoma [1]. It has a predilection for the parotid, with most cases reported in the sixth to the eighth decade with a striking male predilection similar to our patient who reported a parotid mass in a 75-year-old male [1,2]. Our patient has reported a long-standing parotid mass that showed a change in behavior after a superficial parotidectomy with rapid enlargement, facial pain, and autonomic dysfunction characterized by eye tearing similar to most of the reported cases that showed a painless long-standing parotid mass that changes its behavior and shows rapid growth, pain, and facial palsy [2-11]. Radiographically, a large, ill-defined heterogeneously mass measuring around 10 cm with satellite multiple solid cystic masses associated with peripheral enhancement, with fat stranding and thickening of the overlying skin, was seen. Our case was similar to many cases that show irregular heterogeneous mass; however, our case was larger than most reported cases and had peculiar, multiple solid cysts that were not seen in most reported cases [2-11].

Microscopically, our case revealed an area of pleomorphic adenoma presented with a giant collagen Rosset. The malignant component arose from a dysplastic glandular component. A heterogonous malignant epithelial and mesenchymal element was also seen, with a chondrosarcoma element associated with a focal osteoid formation. Squamous cell carcinoma, epithelial myoepithelial carcinoma, and myoepithelial carcinoma formed the epithelial component. An incidental finding in our case was the presence of a solid sheet of Langerhans cells with coffee bean-shaped nuclei and nuclear grooving admixed with eosinophils, which were positive to S100 and CD1a, and BRAF. 

Langerhans cell histiocytosis (LCH) is considered the most common histiocytic disease, characterized by the proliferation of langerin-positive histiocytes (CD207+) with infiltration of inflammatory cells. It can be a localized lesion or involve multiple organs, with the latter showing a more aggressive clinical course [15]. It could arise in any organ, with a tendency for bone, skin, lung, and pituitary glands. Clinical presentation varies from an indolent lesion with single system involvement to a more aggressive lesion that involves multiple organs [15]. Recently, a somatic mutation in the MAPK pathway with 57% of cases showed the BRAF V600E mutation [15]. Langerhans cells are reported to be associated with multiple tumors in different body organs. In some cases, a second malignancy develops after LCH, and it is thought that therapy of LCH induces the second malignancy [18,19]. For others, clonal relationships and genetic predisposition were postulated in malignant cases diagnosed before and concurrent to LCH [19-21]. Our case is unique, as we are reporting a rare entity incidentally found adjacent to a focal LCH. This is the fourth case that reports the presence of LCH adjacent to a salivary gland lesion and the first to report a concurrent LCH with malignant salivary gland neoplasm [16,17,22]. 

Carcinosarcoma ex pleomorphic adenoma is usually managed surgically with adjuvant chemotherapy, adjuvant radiotherapy, or adjuvant chemoradiation; however, all treatment modalities yield a dismal prognosis with tumor progression or death from the disease, with rare cases reporting no tumor recurrence. However, these cases report no follow-up period or a minimum follow-up period [2-4,6-8]. Similarly, our case was surgically removed followed by chemoradiation; however, the lesion progressed and showed metastasis to the lung.

## Conclusions

We herein report the first case report of a concurrent carcinosarcoma ex pleomorphic adenoma with Langerhans cell histiocytosis. Our case shows a long-standing parotid lesion that showed rapid enlargement after surgical intervention; unfortunately, the tumor progressed with the therapy.
